# Genomic Epidemiology of Carbapenem- and Colistin-Resistant *Klebsiella pneumoniae* Isolates From Serbia: Predominance of ST101 Strains Carrying a Novel OXA-48 Plasmid

**DOI:** 10.3389/fmicb.2020.00294

**Published:** 2020-02-21

**Authors:** Mattia Palmieri, Marco Maria D’Andrea, Andreu Coello Pelegrin, Caroline Mirande, Snezana Brkic, Ivana Cirkovic, Herman Goossens, Gian Maria Rossolini, Alex van Belkum

**Affiliations:** ^1^bioMérieux, Data Analytics Unit, La Balme-les-Grottes, France; ^2^Department of Biology, University of “Tor Vergata”, Rome, Italy; ^3^Department of Medical Biotechnologies, University of Siena, Siena, Italy; ^4^bioMérieux, R&D Microbiology, La Balme-les-Grottes, France; ^5^Institute for Laboratory Diagnostics Konzilijum, Belgrade, Serbia; ^6^Institute of Microbiology and Immunology, Faculty of Medicine, University of Belgrade, Belgrade, Serbia; ^7^Laboratory of Medical Microbiology, Vaccine and Infectious Disease Institute, University of Antwerp, Antwerp, Belgium; ^8^Microbiology and Virology Unit, Florence Careggi University Hospital, Florence, Italy; ^9^Department of Experimental and Clinical Medicine, University of Florence, Florence, Italy

**Keywords:** *bla*_OXA–48_, *K. pneumoniae*, colistin, *mgrB*, Serbia, ST101, WGS

## Abstract

*Klebsiella pneumoniae* is a major cause of severe healthcare-associated infections and often shows MDR phenotypes. Carbapenem resistance is frequent, and colistin represents a key molecule to treat infections caused by such isolates. Here we evaluated the antimicrobial resistance (AMR) mechanisms and the genomic epidemiology of clinical *K. pneumoniae* isolates from Serbia. Consecutive non-replicate *K. pneumoniae* clinical isolates (*n* = 2,298) were collected from seven hospitals located in five Serbian cities and tested for carbapenem resistance by disk diffusion. Isolates resistant to at least one carbapenem (*n* = 426) were further tested for colistin resistance with Etest or Vitek2. Broth microdilution (BMD) was performed to confirm the colistin resistance phenotype, and colistin-resistant isolates (*N* = 45, 10.6%) were characterized by Vitek2 and whole genome sequencing. Three different clonal groups (CGs) were observed: CG101 (ST101, *N* = 38), CG258 (ST437, *N* = 4; ST340, *N* = 1; ST258, *N* = 1) and CG17 (ST336, *N* = 1). *mcr* genes, encoding for acquired colistin resistance, were not observed, while all the genomes presented mutations previously associated with colistin resistance. In particular, all strains had a mutated MgrB, with MgrB^C28S^ being the prevalent mutation and associated with ST101. Isolates belonging to ST101 harbored the carbapenemase OXA-48, which is generally encoded by an IncL/M plasmid that was no detected in our isolates. MinION sequencing was performed on a representative ST101 strain, and the obtained long reads were assembled together with the Illumina high quality reads to decipher the *bla*_OXA–__48_ genetic background. The *bla*_OXA–__48_ gene was located in a novel IncFIA-IncR hybrid plasmid, also containing the extended spectrum β-lactamase-encoding gene *bla*_CTX–M–15_ and several other AMR genes. Non-ST101 isolates presented different MgrB alterations (C28S, C28Y, K2^∗^, K3^∗^, Q30^∗^, adenine deletion leading to frameshift and premature termination, IS*5*-mediated inactivation) and expressed different carbapenemases: OXA-48 (ST437 and ST336), NDM-1 (ST437 and ST340) and KPC-2 (ST258). Our study reports the clonal expansion of the newly emerging ST101 clone in Serbia. This high-risk clone appears adept at acquiring resistance, and efforts should be made to contain the spread of such clone.

## Introduction

*Klebsiella pneumoniae* has emerged as one of the most challenging antibiotic-resistant pathogens, since it can cause a variety of infections, including pneumonia and bloodstream infections, and exhibits a remarkable propensity to acquire antimicrobial resistance (AMR) traits. In particular, carbapenem-resistant *K. pneumoniae* (CRKP) are challenging pathogens due to the limited treatment options, high mortality rates, and potential for rapid dissemination in health care settings (Paczosa and Mecsas, 2016).

Treatment options for CRKP infections are usually limited to aminoglycosides, tigecycline, fosfomycin, and colistin. Novel β-lactam-β-lactamase inhibitors combinations, such as ceftazidime-avibactam and meropenem-vaborbactam, have represented a major breakthrough for treatment of some CRKP (e.g., those producing KPC-type and OXA-48-like enzymes), but unfortunately they do not cover strains producing metallo-carbapenemases (Bassetti et al., 2018). Colistin, despite its nephrotoxicity and neurotoxicity, remains a key component of some anti-CRKP regimens (Karaiskos et al., 2017).

Colistin resistance (colR) is mainly mediated by modifications of the lipid A moiety of the bacterial lipopolysaccharide (LPS) by addition of positively charged 4-amino-4-deoxy-L-arabinose (LAra4N) and/or phosphoethanolamine (pEtN) residues. A large panel of genes and operons is involved in modifications of the LPS, and mutations conferring colistin resistance have mainly been observed in *mgrB, phoP/phoQ, pmrA/pmrB*, and *crrB* genes (Cheng et al., 2010; Cannatelli et al., 2013, 2014a; Wright et al., 2015). Recently, several plasmid-mediated colistin resistance genes, named *mcr*, encoding pEtN transferases, have also been reported in *E. coli* and other members of Enterobacterales, including *K. pneumoniae* (Sun et al., 2018).

Global dissemination of CRKP is mainly caused by the spread of a few successful clones. Major representatives of these high-risk clonal lineages include the clonal group (CG) 11, CG15, CG307, CG17, CG37, CG101, and CG147 strains. CG258 strains, and in particular those of ST258, are major players in the worldwide spread of KPC-type carbapenemases, and are responsible for 68% of the CRKP outbreaks (Navon-Venezia et al., 2017). CG101 strains harbor different clinically-relevant resistance determinants, such as carbapenemases of the KPC, OXA-48, VIM, and NDM types. This feature, together with their ability to produce biofilm and several additional virulence factors, is likely a major factor in the ecological success of CG101 strains. Indeed, spreading of this clone is on the rise (Navon-Venezia et al., 2017).

Multidrug resistance (MDR) prevalence in clinical isolates of *K. pneumoniae*, including resistance to third-generation cephalosporins, fluoroquinolones and aminoglycosides, may be as high as 50% in Southern Europe, and even higher proportions have been observed in Eastern Europe. In Serbia, in 2016, MDR *K. pneumoniae* accounted for 63% of all *K. pneumoniae* infections in humans, of which 35% were also carbapenem resistant (WHO Regional Office for Europe, 2017). Previous studies reported that NDM-1 was the main *K. pneumoniae*-associated carbapenemase observed in Serbia in the period 2013–2014 followed by OXA-48, while KPC was only sporadically reported (Grundmann et al., 2017; Trudic et al., 2017). Novović et al. (2017) performed a molecular epidemiology study of carbapenem- and colistin-resistant strains from Serbia, showing prevalence of CG258 and CG101 strains, producing NDM-1 and OXA-48 carbapenemases, respectively. However, the proportion of colistin resistance among those isolates was not reported, and the mechanisms of colistin resistance of those isolates were not elucidated (Novović et al., 2017).

In this study, we used whole genome sequencing (WGS) to study the genomic epidemiology and AMR mechanisms of colR *K. pneumoniae* isolates from Serbia, including some representative of the previously mentioned collection as reference to study the dynamic changes of population structure (Novović et al., 2017).

## Materials and Methods

### Bacterial Isolates and Susceptibility Testing

In the period between November 2013 and May 2017, *K. pneumoniae* isolates were obtained from routine microbiological cultures of clinical samples (e.g., urine, blood, skin, bronchial aspirate) from seven Serbian medical centers distributed in five Serbian cities (Niš, Novi Sad, Belgrade, Kraljevo, and Subotica). Bacteria were not isolated by the authors but provided by the respective medical centers. Therefore, an ethics approval was not required as per institutional and national guidelines and regulations. Information about patients antimicrobial treatment were not available. Identification at the species level was performed by Vitek2 (bioMérieux, Marcy-l’Etoile, France), and carbapenem susceptibility was determined by disk diffusion and interpreted according to the EUCAST breakpoints (EUCAST, 2019). Isolates non-susceptible to at least one carbapenem (ertapenem, meropenem, and imipenem) were tested for colistin resistance by Vitek2 or Etest (bioMérieux, Marcy-l’Etoile, France) according to manufacturer’s instructions (note that the warning by EUCAST about colistin susceptibility testing was only issued in July 2016, and for this reason the above methods were used for colistin susceptibility testing of the isolates collected in this study). Antimicrobial susceptibility testing of the colR isolates was performed using the Vitek2 automated system, and results were interpreted according to EUCAST breakpoints (EUCAST, 2019). Colistin minimum inhibitory concentrations (MICs) were confirmed using the broth microdilution method performed according to the CLSI guidelines (CLSI, 2019) and interpreted by using the EUCAST breakpoints (EUCAST, 2019). For carbapenems (ertapenem, imipenem, and meropenem), MICs were obtained by using Etests (bioMérieux, Marcy-l’Etoile, France). To note, 25 colR isolates were from the previously described collection by Novović et al. (2017), and were included in this study for comparative purposes.

### Mass Spectrometry Analysis of Lipid A

Preparations of lipid A were obtained as previously described (Kocsis et al., 2017). An aliquot of 0.7 μL of each preparation was spotted on a matrix-assisted laser desorption/ionization–time of flight mass spectrometry (MALDI-TOF MS) sample plate, mixed with an isovolume of norharmane matrix (Sigma-Aldrich, St. Louis, MO, United States) and then air-dried. Samples were analyzed with a Vitek MS instrument (bioMérieux, Marcy-l’Étoile, France) in the negative-ion mode.

### DNA Extraction and Whole Genome Sequencing

Genomic DNA was extracted with the DNeasy UltraClean kit (Qiagen, Hilden, Germany), quantified by using the Qubit fluorometer (Thermo Fisher Scientific, United States) and quality checked by using the 260/280 ratio absorbance parameter as determined by the DS-11 FX + instrument (DeNovix, Wilmington, DE, United States). Sequencing was performed using a NextSeq platform (Illumina, Inc., San Diego, CA, United States) and a 2 bp × 150 bp paired-end approach. Raw data from paired-end sequencing were quality checked with the FastQC tool (v.0.11.6) and assembled with SPAdes (v.3.10.1) (Bankevich et al., 2012). One representative strain (KB-2017-139) was also sequenced with the MinION sequencer (ONT, Oxford, United Kingdom) using an R9.5.1 flow cell and the protocol 1D Genomic DNA by Ligation (SQK-LSK109). Illumina and Nanopore raw data from KB-2017-139 were assembled with a hybrid approach using Unicycler (Wick et al., 2017). Whole genome sequencing data of the 45 clinical isolates have been deposited under BioProject PRJNA449293^1^. The complete sequence of the plasmid pSRB_OXA-48 obtained by Illumina and Nanopore sequencing was deposited on GenBank under accession number MN218814.

### Bioinformatics Analysis

MLST was performed *in silico* by using the tool mlst^2^ and the Pasteur database^3^. BLAST + (2.7.1) was used to detect mutations in genes potentially involved in colistin resistance (*mgrB*, *pmrA/B*, *phoP/Q*, *crrA/B*), and only mutations leading to amino acid variations were considered. For the characterization of colistin resistance mechanisms, strains of CG258, ST101 and ST336 were compared to colistin susceptible reference strains of the same CG, i.e., NJST258_2 (accession no. NZ_CP006918.1), BA33875 (NEWA00000000) and MGH-78578 (NC_009648.1), respectively. Phylogenetic relatedness was investigated with the parsnp tool (v1.2) (Treangen et al., 2014) by using default parameters and the strain NTUH-K2044 (accession no. NC_012731.1) as reference. The phylogenetic tree obtained was visualized with the online tool iTol (Letunic and Bork, 2016). The ABRicate tool^4^ was used to detect acquired AMR genes using the ResFinder database (Zankari et al., 2012), while plasmid replicons were predicted by PlasmidFinder (Carattoli et al., 2014). Kaptive was used for the capsular type detection (Wyres et al., 2016). Comparative analysis of plasmids was performed with BLAST Ring Image Generator (Alikhan et al., 2011) and Easyfig (Sullivan et al., 2011).

For the comparative genomic analysis of ST101 isolates, on 31 October, 2018 all the *K. pneumoniae* genomes available on NCBI (*N* = 5,820) were downloaded with the ncbi-genome-download tool^5^. MLST was performed and all ST101 (*N* = 195) (Supplementary Table S2) together with ST101 strains from this study were used for phylogenetic investigations by using parsnp and the closed ST101 chromosome from Kp_Goe_121641 (accession no. NZ_CP018735.1) as reference.

## Results

### *Klebsiella pneumoniae* Isolates and Antimicrobial Susceptibilities

In the period between November 2013 and May 2017, a total of 2,298 clinical isolates of *K. pneumoniae* were isolated from patients admitted to seven medical settings located in five Serbian cities. Among those, 426 isolates (18.5%) were non-susceptible to at least one carbapenem by disk diffusion, and were tested for colistin resistance. A total of 45 strains (10.6%) out of this subset showed a colistin resistant phenotype. At the time of the collection, colistin susceptibility testing was routinely performed with the Vitek2 instrument or Etest, although these methods had several limitations (Tan and Ng, 2007). Thus, the number of colR isolates may be underestimated.

All the strains were confirmed as colistin resistant by the broth microdilution method (considering the EUCAST susceptibility breakpoint of 2 mg/L) with MICs that ranged between 8 and 32 mg/L (Supplementary Table S1). Etest results for carbapenemes showed that all the strains were resistant to ertapenem, while meropenem and imipenem had susceptibility rates of 93.3 and 91.1%, respectively. Vitek2 results showed that none of the fluoroquinolones, penicillins combined with β-lactamase inhibitors and cephalosporins (including cefoxitin and the 4th generation cephalosporin cefepime) were effective against the 45 colR isolates. Conversely, amikacin (86% susceptibility) and trimethoprim/sulfamethoxazole (78% susceptibility) were the most active agents together with imipenem and meropenem (Supplementary Table S1).

### Genomic Epidemiology

**FIGURE 1:**
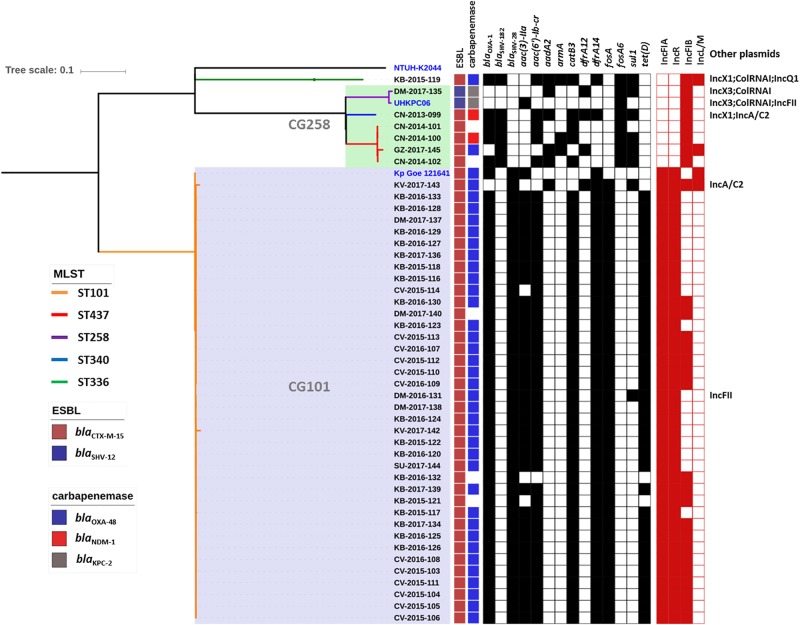
Phylogenetic tree of the colR *Klebsiella pneumoniae* isolates from Serbia. For each isolate, the medical setting (CN, Clinical center of Niš, Niš; CV, Clinical center of Vojvodina, Novi Sad; KB, Konzilijum, Belgrade; DM, University hospital center “Dr. Dragiša Mišovic-Dedinje,” Belgrade; KV, The General hospital “Studenica,” Kraljevo; GZ, The Institute of Public health of Belgrade, Belgrade; SU, General Hospital Subotica, Subotica), the year of isolation and the sample number are reported. Colored nodes indicate MLST, while the presence/absence of ESBLs, carbapenemases, resistance genes (black) and plasmid replicons is indicated by filled boxes.

Genome sequence data were used to investigate the population structure of the colR *K. pneumoniae* strains circulating in Serbia. Five different STs were detected among the investigated collection (ST101, ST437, ST258, ST336, and ST340), with the majority of strains belonging to ST101 (*N* = 38) or CG258 (ST258, *N* = 1; ST340, *N* = 1 and ST437, *N* = 4) (Figure 1). The remaining strain belonged to CG17 and was typed as ST336. Isolates of ST101 were closely related to each other [single nucleotide polymorphism (SNP) variation: 5–893, mean 107, median 61], with only two of them (i.e., KV-2017-142 and KV-2017-143) having more than 200 SNPs when compared to other ST101 isolates and to each other. The ST101 isolates were detected in all the cities involved in this study, except Niš, thus demonstrating the endemicity at the national level of this clone. Moreover, there was not a clear clustering of isolates obtained from different hospitals, suggesting inter-hospital cross infections.

**FIGURE 2:**
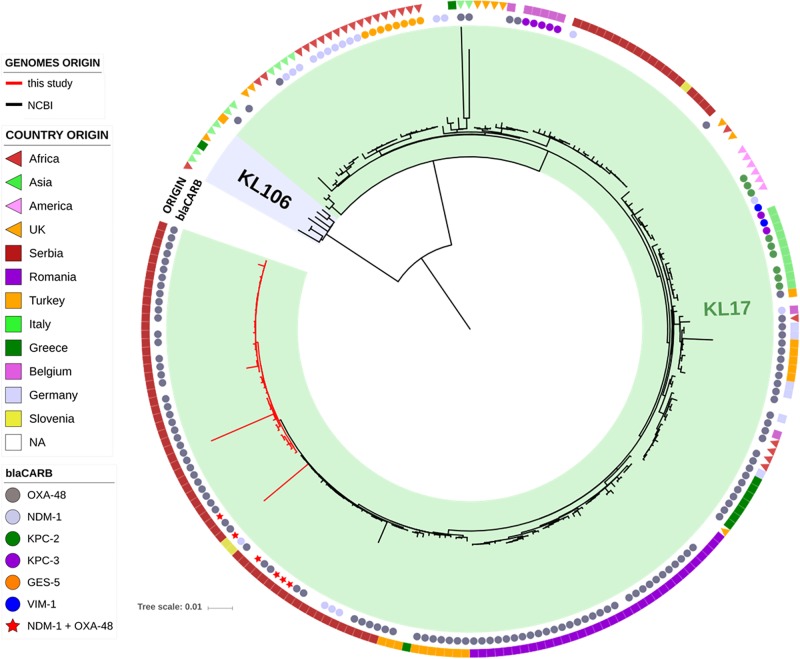
Phylogenetic tree of the ST101 *K. pneumoniae* isolates from this study (red lines) in comparison to ST101 isolates retrieved from NCBI (black lines). The two types of capsular polysaccharides (KL17 and KL106) are indicated by colored ranges. Two datasets are also present, indicating the type of carbapenemase (inner circle) and the country of origin (outer circle).

The genomes of the ST101 Serbian isolates were compared with 195 ST101 genomes available in the INSDC databases, and their phylogenetic relation is showed in Figure 2. Strains from our study (red lines) cluster together in the tree in a well-defined branch containing other strains from Serbia, Slovenia, Turkey and Greece. Overall, the number of SNPs among all analyzed ST101 isolates ranged between 1 and 1,547 (mean 195, median 135), and two major lineages within this group can be observed. The majority of SNPs separating these two lineages fell in the gene cluster, and this was consistent with the previous observations that strains of ST101 are characterized by two different K-loci, KL17 and KL106, associated with *wzi* alleles 137 and 29, respectively (Roe et al., 2019). While KL17 is prevalent among ST101 strains, KL106 is less frequent but, interestingly, it is the second most abundant capsular variant of CG258 (Wyres et al., 2015), reinforcing the hypothesis that capsular exchange in *K. pneumoniae* is a common event (Chen et al., 2014; Bowers et al., 2015).

All non-ST101 isolates (excluding KB-2015-119) were part of a single monophyletic subclade within the CG258 (Bowers et al., 2015) and produced different carbapenemases or were carbapenemase negative (Figure 1), while the remaining isolate of ST336 was a OXA-48-producer and harbored the KL25 capsular type.

### Colistin Resistance Mechanisms

No *mcr* genes were observed in the genomes of the colR isolates. Conversely, all of them showed alterations in the PhoP/PhoQ regulator *mgrB* gene. These alterations were mainly SNPs, with the majority of ST101 isolates from this study characterized by the mutation MgrB^C28S^ (*N* = 37; 97.4%). Although different substitutions of the cysteine amino acid at position 28 have already been described (e.g., MgrB^C28F^ and MgrB^C28Y^), and their role in colistin resistance has been experimentally demonstrated (Cannatelli et al., 2014b; Olaitan et al., 2014; Cheng et al., 2015; Wright et al., 2015), the MgrB^C28S^ is first described here. This cysteine residue has been previously shown to be involved in a key disulfide bond relevant to MgrB function (Lippa and Goulian, 2012), thus its substitution by Serine or by any other amino acid is expected to interfere with the ability to repress PhoQ, leading to the overexpression of the *pmrHFIJKLM* operon and to a colistin resistance phenotype. The isolate CN-2013-099, belonging to ST340, displayed the previously studied MgrB^C28Y^ substitution (Cheng et al., 2015). Different mutations leading to premature stop codons were MgrB^K2*^ in the ST101 isolate KV-2017-143, firstly described here, MgrB^K3*^ in the ST437 isolate GZ-2017-145 (Nordmann et al., 2016) and MgrB^Q30*^ in the ST336 strain KB-2015-119 (Nordmann et al., 2016). The ST258 isolate was characterized by an insertion sequence of the family IS*5* which interrupted the *mgrB* gene at nucleotide 75. Disruption of the *mgrB* gene by insertion sequences has been shown as a common mechanism of colistin resistance in KPC harboring strains (Cannatelli et al., 2014b). Three ST437 strains were characterized by an adenine deletion within the polyadenine region present from nucleotide 4 to 9 in *mgrB*, resulting in a frameshift mutation. Collectively, the results of these analyses demonstrated that all colistin resistant strains investigated in this study were characterized by genetic alterations in the *mgrB* gene.

Other genetic alterations potentially involved in colistin resistance were: PmrA^E57G^ (KB-2015-119, ST336), PmrB^T157P^ (CCV-2015-105, ST101) and PhoQ^V446G^ (CCDM-2017-135, ST258). Among these, only PmrB^T157P^ was previously reported, and its role in reducing colistin susceptibility was demonstrated (Jayol et al., 2014). Accordingly, the ST101 isolate CV-2015-105 having PmrB^T157P^ together with MgrB^C28S^, showed a colistin MIC 1- to 2-fold higher than isogenic strains carrying only MgrB^C28S^.

Mass spectrometry of lipid A was performed on a subset of isolates representative of the different alterations potentially involved in colistin resistance. Compared to the colistin susceptible reference ATCC11296 strain, colR isolates showed an additional peak at 1,971 m/z resulting from the addition of a 4-amino-4-deoxy-L-arabinose moiety (131 m/z) to lipid A (peak at 1,840 m/z), as previously reported (Leung et al., 2017) (results not shown). This supports the role of the observed mutations in the overexpression of the *pmrHFIJKLM* operon and consequent lipid A modification, leading to reduced colistin interactions. Moreover, no addition of pEtN moieties to lipid A were observed, consistently with the absence of *mcr*-like genes (Liu et al., 2017).

To note, our findings concerning MgrB alterations differ from those previously reported by Novović et al. (2017), as they did not detect significant MgrB alterations for most of the isolates. This underline the importance of using well-characterized colistin susceptible reference isolates, as the one used in the mentioned study was not characterized with reference methods for colistin susceptibility testing (Mirovic et al., 2012).

### Other Antibiotic Resistance Mechanisms

All strains were positive for an ESBL-encoding gene, with *bla*_CTX–M–__15_ harbored by all strains except the only ST258, which carried a *bla*_SHV–__12_ gene. Analysis of the *ompK35* gene, encoding a major outer membrane protein, showed that all non-ST258 strains had deletions leading to frameshift and premature stop codons, while the *ompK36* gene was intact in all the genomes. Outer membrane impermeability most likely explains resistance to cefoxitin (a cephamycin) and to ertapenem for those isolates negative for a carbapenemase encoding gene (Ardanuy et al., 1998). Two ST437 and the ST336 isolate harbored the 16S rRNA methylase gene *armA*, which confers high level resistance to aminoglycosides. Several other AMR genes were observed for the following antimicrobial classes: aminoglycosides (presence of *aac*-, *aad*-, *aph*-, and *ant*-type modifying enzymes), fluoroquinolones (*oqxAB*, *qnrB1*, *aac(6′)-Ib-cr*, *parC^*S*80*I*^*, *gyrA^*S*83*Y–S*83*I–D*87*G–D*87*N*^*), phenicol (*floR*, *catA1* and *catB4* genes), sulfonamide (*sul1* and *sul2* genes), tetracycline (*tetA* and *tetD* genes) and trimethoprim (*dfrA*).

### Novel IncR/IncFIA OXA-48 Plasmid Within ST101 Isolates

The production of OXA-48 was at the basis of carbapenem resistance in the *K. pneumoniae* of ST101 analyzed in this study. For this reason, we deeply investigated the genetic context of this gene. Spreading of the *bla*_OXA–__48_-encoding gene among *Enterobacterales* is mainly related to the dissemination of a single ∼62-kb IncL/M-like conjugative plasmid (Poirel et al., 2012). However, PlasmidFinder analysis did not detect any IncL/M replicon among ST101 isolates from Serbia. Therefore, MinION sequencing was performed on one representative strain (KB-2017-139) with the aim to fully characterize the genomic background of the *bla*_OXA–__48_ gene.

**FIGURE 3:**
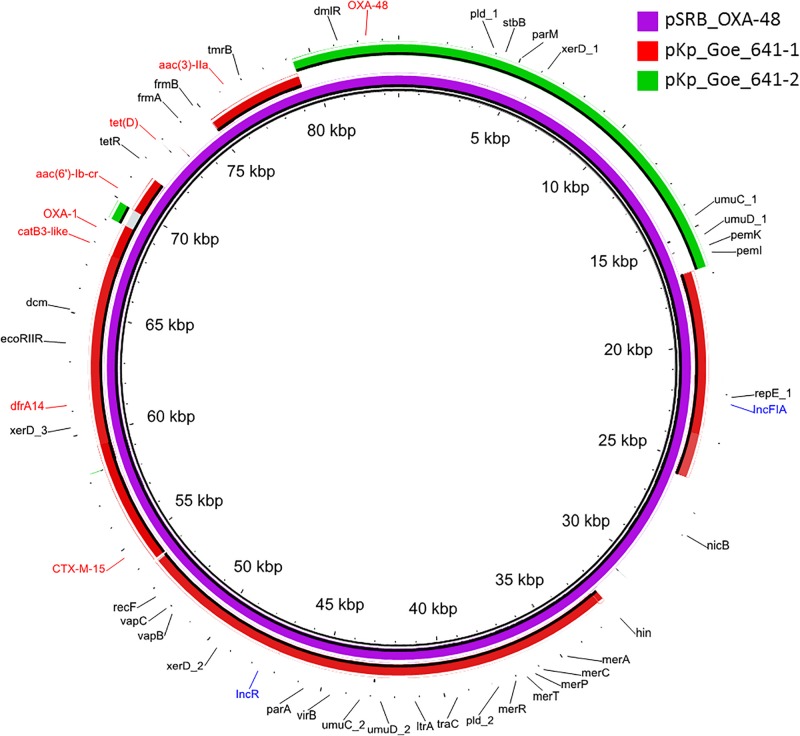
BLAST ring image generator output of the OXA-48 plasmid pSRB_OXA-48 from the ST101 isolate KB-2017-139 (violet) against the two major plasmids from the ST101 isolate Kp_Goe_1216141 (pKp_Goe_641-1, in red and pKp_Goe_641-2 in green). Only identities > 95% are indicated. Antimicrobial resistance genes are indicated in red, plasmid replicons in blue and all other genes in black.

**FIGURE 4:**
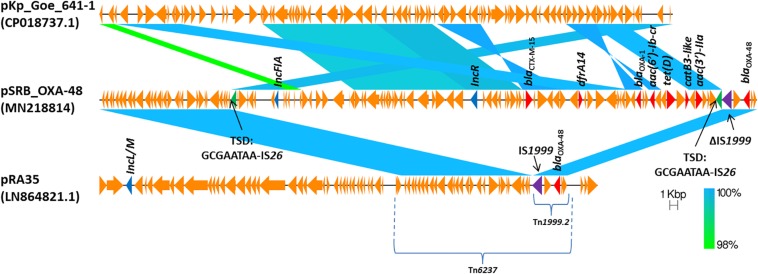
Comparison of plasmids pSRB_OXA-48, pKpGoe_641-1 and pRA35. Antimicrobial resistance genes, plasmid replicons and mobile elements are also indicated. TSD: target site duplication.

The *bla*_OXA–__48_ gene was located on a plasmid of 83,654 bp, named pSRB_OXA-48, carrying both the IncR and the IncFIA type replicons, the *bla*_CTX–M–__15_ and several other AMR genes (*tet(D)*, *aac(6′)-Ib-cr*, *bla*_OXA–__1_, *catB3-like*, *aac(3′)-IIa* and *dfrA14*). A BLAST analysis showed that pSRB_OXA-48 is a hybrid plasmid composed by (i) a fragment having 99.7% identity with the IncFIA-IncR pKp_Goe_641-1 plasmid (CP018737.1) and carrying the *bla*_CTX–M–__15_ gene and several other AMR genes [*aac(3)-IIa*, *catB3*, *bla*_OXA–__1_, *aac(6′)-Ib-cr*, *aac(6′)-Ib*, *ant(3′′)-Ia*, *bla*_OXA–__9_, *bla*_TEM–1A_, *dfrA14*], and (ii) a fragment identical to the IncL/M plasmid pKp_Goe_641-2 (CP018736.1) carrying the *bla*_OXA–__48_ gene (Figure 3). Both these plasmids have been described in *K. pneumoniae* strain Kp_Goe_121641 (accession no. NZ_CP018735.1), isolated from a refugee from North Africa hospitalized in Germany, in 2013. The latter strain belongs to ST101 and has a median of 142 SNPs (minimum 134, maximum 601) compared to the Serbian ST101 isolates from this study. Collectively these results suggest that pSRB_OXA-48 likely originated by recombination events between two plasmids within an ST101 strain related to Kp_Goe_121641. In order to elucidate the recombination mechanisms at the origin of pSRB_OXA-48, we compared this plasmid to pKp_Goe_641-1 and to pRA35 (LN864821.1), an IncL/M plasmid similar to pKp_Goe_641-2 but with an intact structure of the transposon Tn*6237* carrying *bla*_OXA–__48_ (Beyrouthy et al., 2014) (Figure 3). A detailed analysis showed that pSRB_OXA-48 contained a copy of Tn*6237* which was disrupted by a IS*26* composite transposon of 73.7 kbp sharing similarity with pKp_Goe_641-1. This hypothesis was corroborated by the presence of 8-bp target site duplication sequences (5′-GCGAATAA-3′) flanking the composite transposons regions (Figure 4). The results of reads-mapping performed against pSRB_OXA-48 using Illumina short-reads from the other ST101/OXA-48 strains was consistent with the presence of a pSRB_OXA-48-related plasmid in all the ST101/OXA-48 isolates. Non-ST101 OXA-48 strains (ST336 KB-2015-119 and ST437 GZ-2017-145) had the IncL/M replicon, while lacking the IncFIA and IncR replicons, suggesting that the *bla*_OXA–__48_ gene was located in a classic IncL/M plasmid and not in a pSRB_OXA-48-like plasmid (Figure 1).

## Discussion

This study exploited WGS to characterize a collection of colR CRKP isolates obtained from seven medical settings and five Serbian cities over a nearly 4-year period. Results showed that all the isolates presented alterations in the PhoP/PhoQ regulator MgrB, confirming its major role in colR in *K. pneumoniae*. Lipid A alterations associated with colR were also studied with MALDI-TOF MS. The analysis revealed the addition of a 4-amino-4-deoxy-L-arabinose moiety to lipid A, but no addition of pEtN moieties, for all isolates tested. These results support the role of the MgrB mutations in colistin resistance, and also confirm the absence of *mcr*-like genes.

The predominant ST observed was ST101, an emerging high-risk clone detected worldwide and associated with different carbapenemases and high mortality (Navon-Venezia et al., 2017; Can et al., 2018). In a recent European survey of CRKP isolates, including 244 hospitals in 32 countries, four major clonal lineages accounted for roughly 70% of the carbapenemase-producing isolates, including ST 11, 15, 101, 258/512 and their derivatives (David et al., 2019). The first ST101 strain from Serbia was isolated in 2013, and coproduced the OXA-48 and the NDM-1 carbapenemases (Seiffert et al., 2014). Most of the colR ST101 from this study were carbapenemase-producers, and OXA-48 was the only carbapenemase expressed. ST101/OXA-48 has been frequently reported, and in an 11-year epidemiology study of OXA-48 producers among European and north- African countries, a quarter of the OXA-48 *K. pneumoniae* isolates belonged to ST101 (Potron et al., 2013). Outbreaks of ST101/OXA-48 were also described, with reports from Spain (Pitart et al., 2011; Cubero et al., 2015), Algeria (Loucif et al., 2016), Czech Republic (Skálová et al., 2016) and Greece (Avgoulea et al., 2018). The challenging phenotypic detection of OXA-48 carbapenemases and the rapid horizontal transfer of OXA-48-encoding plasmids favor hospital outbreaks linked to patient transfer (Skálová et al., 2016) and draw attention to the need for continuous and meticulous surveillance, as well as timely investigation.

The *bla*_OXA–__48_ gene spread is mainly related to the dissemination of a single ∼62-kb IncL/M-like conjugative plasmid that does not carry additional resistance determinants (Poirel et al., 2012). Conversely, ST101/OXA-48 isolates from this study carried a novel hybrid plasmid (pSRB_OXA-48) with replicons IncR and IncFIA and encoding OXA-48, the CTX-M-15 ESBL and several other AMR genes. Such plasmids confer an MDR phenotype which limits the use of most β-lactams, including carbapenems. In fact, even if most isolates (91%) were susceptible to imipenem, carbapenems have been proven to be not effective in an *in vivo* murine model (Wiskirchen et al., 2014). Moreover, there have been a number of case reports and series describing treatment failures with carbapenem-containing regimens in the treatment of OXA-48-producing bacterial infections (Stewart et al., 2018). Ceftazidime-avibactam may represent an effective alternative against such isolates, as previously reported (Kazmierczak et al., 2018).

Similarities among the Serbian ST101 strains, supported by the limited number of SNPs observed and the presence of the same alteration in the *mgrB* gene, suggest a clonal expansion of this clone among Serbian medical settings. This observation underscores the need to strengthen contact precautions for patients diagnosed with or suspected of having CRKP infections to limit the diffusion of colR CRKP of ST101.

Of note, colR ST101 strains have recently been associated with high mortality rates. Indeed, a prospective cohort study showed that among colR isolates, ST101 was found to be a significant independent predictor of patient mortality, with a 30 day patient mortality of 72% (Can et al., 2018).

## Conclusion

This work corresponds to the first genomic investigation of colistin resistance in *K. pneumoniae* isolates from Serbia. The major role of MgrB mutations in colistin resistance in *K. pneumoniae*, observed in strains of CG258, is here confirmed for those of ST101. We also report the full sequence of a novel plasmid, pSRB_OXA-48, conferring MDR phenotype and encoding for the ESBL CTX-M-15 and the carbapenemase OXA-48.

## Data Availability Statement

The datasets generated for this study can be found in the www.ncbi.nlm.nih.gov/bioproject/PRJNA449293.

## Author Contributions

AB, GR, and HG conceived and designed the study. MP performed the Phenotypic and Genomics experiments. MP and MD’A performed the bioinformatics analysis. All authors analyzed the data and contributed to the manuscript.

## Conflict of Interest

MP, AP, CM, and AB are employees of bioMérieux, a company that develops and sells diagnostic tests in the field of infectious diseases. The remaining authors declare that the research was conducted in the absence of any commercial or financial relationships that could be construed as a potential conflict of interest.

## Footnotes

1 www.ncbi.nlm.nih.gov/bioproject/PRJNA449293

2 https://github.com/tseemann/mlst

3 https://bigsdb.pasteur.fr/

4 https://github.com/tseemann/abricate

5 https://github.com/kblin/ncbi-genome-download
